# Super-infection by multiple microorganisms in COVID-19 patients

**DOI:** 10.3389/fmolb.2023.1113969

**Published:** 2023-03-13

**Authors:** Andrea C. Gomez, Tamin Ortiz, Angélica Valenzuela, Rocío Egoávil-Espejo, Rosario Huerto-Huanuco, Joseph A. Pinto, Jose Lagos, Joaquim Ruiz

**Affiliations:** ^1^ Centro de Investigación Básica y Translacional, AUNA IDEAS, Lima, Peru; ^2^ Servicio de Microbiología y Biología Molecular, Laboratorios AUNA, Lima, Peru; ^3^ Grupo de Investigación en Dinámicas y Epidemiología de la Resistencia a Antimicrobianos—“One Health”, Universidad Científica del Sur, Lima, Peru

**Keywords:** COVID-19, co-infection, antimicrobial resistance, hospitalization time, Latin America

## Abstract

**Introduction:** This study aimed to describe the clinical characteristics of patients with COVID-19 co-infected with multiple multidrug-resistant bacteria.

**Methods:** Patients hospitalized in the AUNA network between January and May 2021, diagnosed with COVID-19 and at least two other infecting microorganisms, were retrospectively included in the analysis. Clinical and epidemiological data were extracted from clinical records. The susceptibility levels of the microorganisms were determined using automated methods. Antibiotic resistance was established among infecting bacteria accounting for ≥5 isolates.

**Results:** A total of 27 patients (21 male and 6 female patients) met the inclusion criteria, with a maximum of eight co-infecting bacteria or fungi during admission time. Seven patients (25.9%) died, with a higher but not significant lethality among women (50% vs. 19.0%). A total of 15 patients presented at least one established comorbidity, with hypertension being the most frequent. The time elapsed between COVID-19 diagnosis and hospital attendance was 7.0 days, with that of patients with a fatal outcome being longer than that of living patients (10.6 vs. 5.4). Up to 20 different microorganisms were isolated, with *Pseudomonas aeruginosa* being the most common (34 isolates). In general, antibiotic resistance levels were high, especially in *Acinetobacter baumannii* isolates, with resistance levels of 88.9% to all antimicrobial agents tested, except colistin (0%).

**Conclusion:** In conclusion, the present results show the presence of multiple microorganisms that co-infect COVID-19 patients. When fatal outcome rates are in the range of other reports, the presence of a series of multidrug-resistant microorganisms is of concern, showing the need to reinforce control measures to limit the expansion of almost untreatable microorganisms.

## 1 Introduction

The COVID-19 pandemic has challenged healthcare systems around the world, with particular virulence in low- and middle-income countries. Currently, there are >636 million positive cases of COVID-19 and 6.4 million confirmed deaths worldwide (Our World, 2022a; World Health Organization, 2022). Regarding Peru, there are 4.1 million positive patients and more than 215,000 deaths caused by COVID-19 (Ministerio de Salud, 2022; World Health Organization, 2022). Currently, after the efforts of several pharmaceutical industries and the Peru government, Peru fully vaccinated >28 million persons, which is around 84% of the population (Our World, 2022b; World Health Organization, 2022).

The men had a rate of hospitalization related to COVID-19 of around 60%, while that of women was around 40% (Docherty et al., 2020). The appearance of symptoms has been described to start around 4 days prior to hospitalization (Docherty et al., 2020), but this time can be longer in patients who self-medicated due to confidence in the drug consumed and had a false sense of improvement (Zavala-Flores and Salcedo-Matienzo, 2020)**.** Among patients with COVID-19 admitted to intensive care units (ICU), the mechanical ventilation requirement has been reported to reach 87.3% (Grasselli et al., 2020)**.**

Patients infected with COVID-19 admitted to the ICU may contract a nosocomial infection caused by a multidrug-resistant (MDR) microorganism; this challenges the accurate diagnosis, treatment, and prognosis of the infection and largely increases mortality (Vijay et al., 2021). These superinfections might be favored by different factors; thus, it has been described as lymphocyte depletion and a subsequent immunosuppression degree (Liu et al., 2021). In this sense, increases in IL-6 and MCP-1, both considered as immunosuppressive cytokines, have been proposed as predictors of death by COVID-19 in hospitalized patients (Pons et al., 2021).

Currently, data on bacterial co-infections in patients admitted due to SARS-CoV-2 are scarce in Peru, this being a relevant knowledge gap. The common high levels of antimicrobial resistance described among the community and nosocomial microorganisms in the country (Levy-Blitchtein et al., 2018; Flores-Paredes et al., 2021; Alcedo et al., 2022) and the high levels of antibiotic consumption during the pandemic (Zavala-Flores and Salcedo-Matienzo, 2020; Moyano et al., 2022) have probably contributed to creating even more multidrug-resistant pathogenic bacteria (Ruiz, 2021). Although these findings highlight the need to fill in this gap of knowledge, it is strongly challenged by the exceptional situation of living during the pandemic, resulting in an extreme stress over the healthcare system, which might have contributed to the non-inclusion of several data on records.

In this scenario, the present study aimed to retrospectively describe the clinical characteristic of patients co-infected with COVID-19 and multiple microorganisms.

## 2 Materials and methods

### 2.1 Patients

Patients hospitalized in the AUNA clinic network from January to May 2021 with a positive molecular test of COVID-19 and a diagnosis of more than one co-infecting microorganism were included in the analysis. A variety of samples, including respiratory tract (bronchial, pharyngeal, tracheal, and sputum) samples, blood, and urine, among other samples, were collected by specialized personnel, refrigerated, and directly transferred to the laboratory and processed within the frame of standard clinical practice. Following internal protocols, nasal and perianal swabs were taken and cultured at the time of admission. No patient accomplished the criteria for other bacterial culture.

Samples related to COVID-19 diagnosis were processed in a biosafety level 3 laboratory, while other microorganisms were managed within a 2A level biosafety cabinet in a biosafety level 2 laboratory.

### 2.2 Clinical and demographic characteristics

Demographic data were obtained from internal patient medical records, including sex, age, hospitalization stay, and outcome. Information about microorganisms, samples, and antibiotic treatment was considered in each group. On the other hand, data from patients who needed additional oxygen requirements presented comorbidities, and antimicrobial resistance levels were extracted from hospital records. Infecting microorganisms were recorded in the order of temporal isolation (for text specifications, this is reported following the nomenclature of first infection, second infection, and successive infections). Infections were considered hospital-acquired infections (HAIs) when they manifested >48 h after admission.

### 2.3 Microorganisms

The identification of bacteria and the antimicrobial susceptibility profile was established using automated tools (VITEK-2, bioMérieux, Marcy l’Etoile, France). Clinical and Laboratory Standards Institute (CLSI) and European Committee on Antimicrobial Susceptibility Testing (EUCAST) breakpoints were considered to determine the susceptibility of *Candida* spp. and *Aspergillus* spp. (CLSI, 2017; European Committee on Antimicrobial Susceptibility Testing, 2022). Antibiotic susceptibility, except colistin, was confirmed and expanded by the disk diffusion assay, according to the guidelines (CLSI, 2021). The MIC for colistin was established, following previously described procedures (Pons et al., 2020). In the absence of specific records, coagulase-negative *Staphylococcus* (CoNS) isolation was considered contamination (Elzi et al., 2012)**.** When the same microorganism was isolated twice or more from the same patient, only the first isolate was assessed for all purposes, except if differences in antibiotic resistance patterns were observed.

### 2.4 Statistical analysis

The data were analyzed using the R program. Significant differences were considered when *p* < 0.05. For analysis purposes, intermediate and resistant isolates were analyzed together and referred to as resistant through the text.

### 2.5 Ethical approval

The study was approved by the IRB of Universidad Científica del Sur (Approval: 074-2020-PRO99), which waived the need for informed consent. Personally identifiable information on the participants was anonymized upon extraction of the relevant data for the study, and patients were coded using numbers (for example, 1, 2, or 3).

## 3 Results

Data obtained from internal electronic medical records presented information from 27 patients co-infected with SARS-CoV-2 and multiple microorganisms during hospitalization. Of these, 21 (77.7%) were male. The age of the patients ranged from 32 to 90 years, with a mean age of 59.8 years. A total of 26 (96.3%) patients needed additional oxygen at any time of admission. Seven patients (25.9%) who required additional oxygen had a fatal outcome. Notably, fatal outcomes were more frequent among women (50.0% vs. 19.0%) but did not reach significance (*p* = 0.2896) (Table 1) All fatal outcomes but one occurred in the hospital setting. The remaining fatal outcome, occurring 37 days after hospital discharge, had no data recorded about death circumstances or new admission to AUNA centers or other hospital settings.

**TABLE 1:** Demographic characteristics of patients.

	N (%)	Age^a^	Ventilation			Death	
			Time^b^	N	%	N	%
Overall	27 (100.0)	59.8	108.6	26	96.4	7	25.9
Male	21 (77.7)	58.5	119.7	20	95.2	4	19.0
Female	6 (22.2)	64.7	69.3	6	100.0	3	50.0

^a^ Median age.

^b^ Median hospitalization time (days).

^c^ Patients needing mechanical ventilation.

**FIGURE 1:**
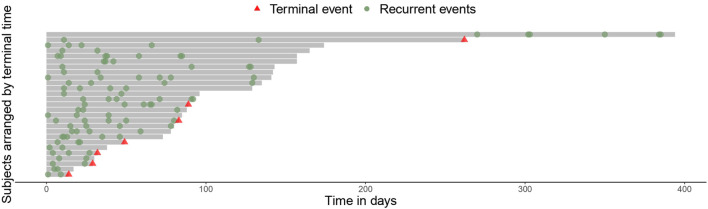
Timeline of the infections.

The mean hospitalization time was 108.6 days, with a maximum stay of 394 days (patient 18); meanwhile, the shorter hospitalization time was 14 days (patient 8). It should be noted that hospitalization time for men was longer than that for women (119.7 days vs. 69.3 days) (Table 1; Figure 1).

All patients but one were diagnosed with COVID-19 before or at hospital admission. In these cases, the time elapsed from the diagnosis of COVID-19 to hospital admission varies from 0 to 38 days, with an overall mean of 7.0 days, which varies from 5.4 to 10.6 days among living and dead patients, respectively, and from 5.9 to 9.8 days when referring to men and women, respectively. The longer time (38 days) was observed in a woman with a fatal outcome. The remaining patients were diagnosed with COVID-19 21 days after admission.

On the other hand, 15 (55.5%) patients had comorbidities, and the most common comorbidity was arterial hypertension (*n* = 5). Obesity was observed in four patients and diabetes mellitus in three patients. Some type of cancer or malignant tumor was presented in two patients. Furthermore, other comorbidities, such as lupus erythematosus, Parkinson’s, and tuberculosis, were observed. Five patients had more than one comorbidity at a time. Of the patients with comorbidities, three (20%) had a fatal outcome (Tables 2, 3).

**TABLE 2:** Patients with a fatal outcome.

Patient	Age	Sex	Time^a^	Comorbidity	Coinfection^b^
5	90	M	32	Hypertension	3
7	63	F	89	---	6
8	79	F	14	---	2
12	76	F	49	Rheumatoid arthritis	3
16^c^	63	M	262	---	3
19	42	M	29	---	2
22	36	M	83	Obesity	5

M, male; F, female.

^a^ Time from admission to fatal outcome.

^b^ Number of coinfecting microorganisms.

^c^ Fatal outcome 37 days after hospital discharge.

**TABLE 3:** Patients with comorbidities.

Patient	Age	Sex	Time^a^	Comorbidity	Outcome
2	55	M	55	Hypertension	A
3	77	F	77	Hypertension and hypothyroidism	A
4	82	M	82	Anemia and diabetes mellitus	A
5	90	M	90	Hypertension	D
9	72	M	72	Prostatic carcinoma	A
10	57	M	57	Tuberculosis	A
11	82	M	82	Diabetes mellitus	A
12	76	F	76	Rheumatoid arthritis	D
13	55	M	55	Obesity	A
14	81	M	81	Diabetes mellitus, hypertension, and Parkinson’s	A
17	46	M	46	Hypertension, obesity, and hypertriglyceridemia	A
18	42	M	42	Obesity	A
20	35	F	35	Lupus erythematosus and fibromyalgia	A
22	36	M	36	Obesity	D
24	74	M	74	Tongue tumor	A

M, male; F, female; A, alive; D, death.

^a^ Hospitalization time.

**FIGURE 2:**
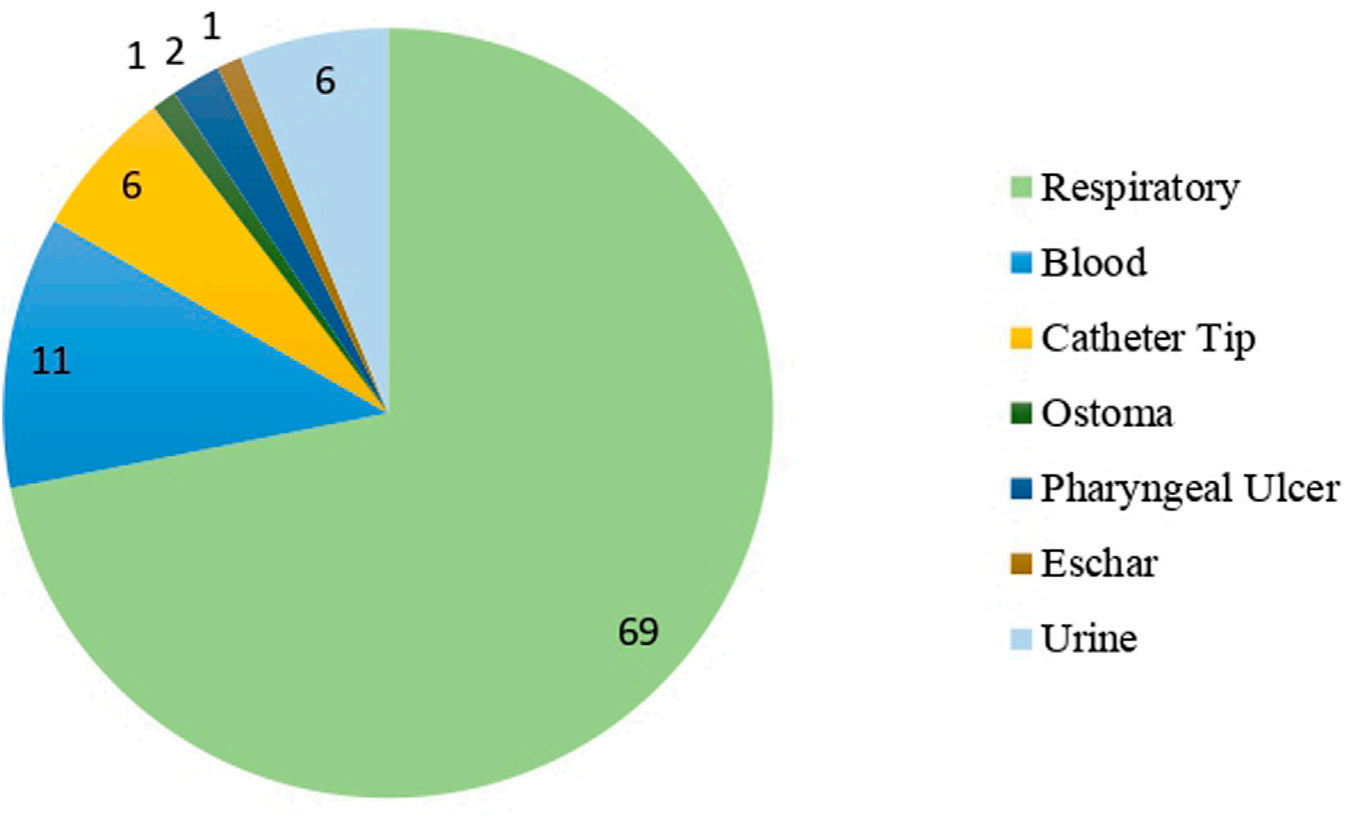
Type of samples used for the diagnosis of patients.

The most frequent samples were respiratory tract samples (69 samples, 60%), including bronchial samples as the most common, but also sputum, tracheal, and pharyngeal samples. Other samples included blood (11 samples, 9.6%), a catheter (6 samples, 5.2%), and urine (6 samples, 5.2%), among others (Figure 2).

**FIGURE 3:**
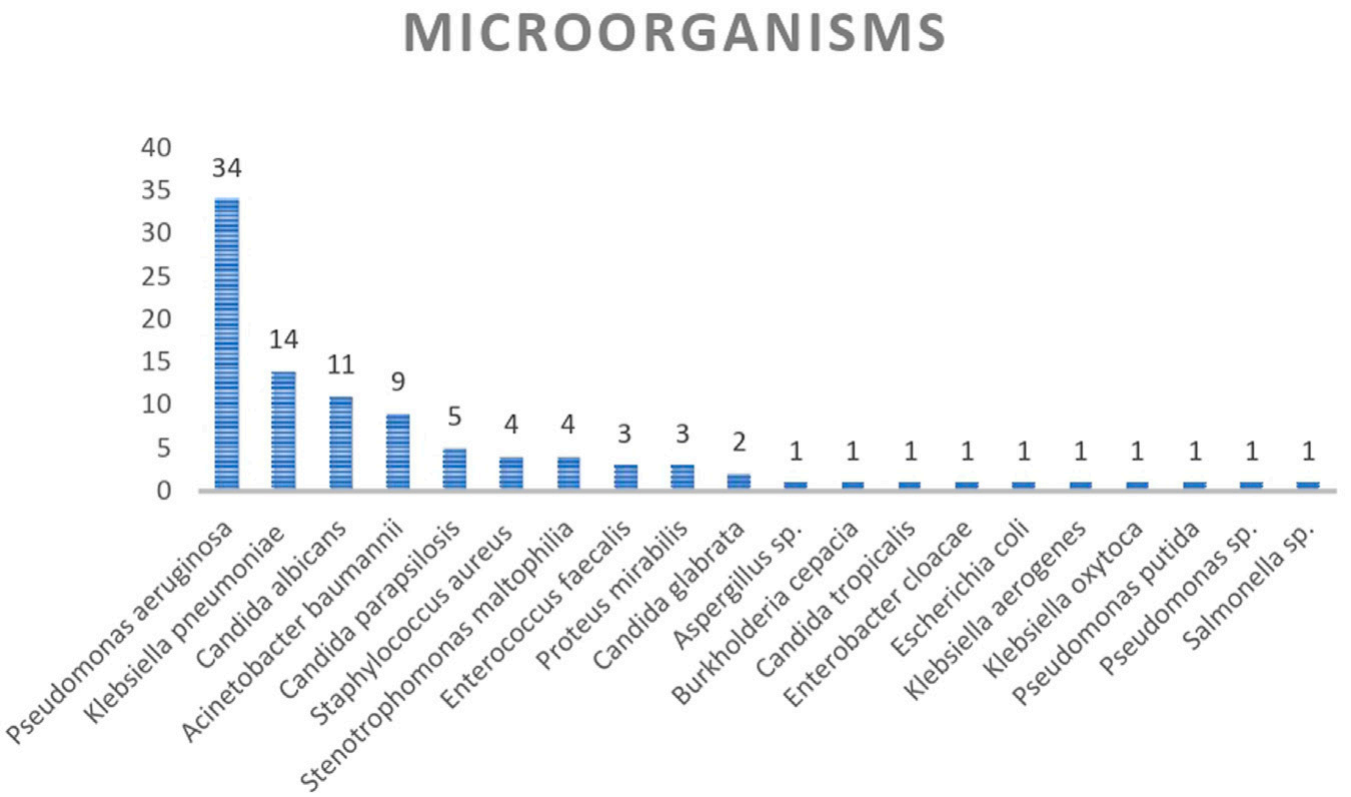
Microorganisms isolated in hospitalized COVID-19 patients with multiple infections.

During the attendance, at least two additional infecting microorganisms were isolated, with a patient presenting eight different additional co-infecting microorganisms during hospitalization. Up to 20 different co-infecting microorganisms were isolated. The most common bacterial pathogen was *Pseudomonas aeruginosa,* accounting for 34 isolates, followed by *Klebsiella pneumoniae* ssp. *pneumoniae* with 14 isolates and *Acinetobacter baumanii* complex with nine isolates. Furthermore, the presence of *Candida albicans* was confirmed in 11 isolates (Figure 3). The first concomitant bacterial infection usually occurs after 4 days after hospitalization, with only five infections accounting for days 0 and 2, thereby qualifying as community-acquired infections. All subsequent co-infections are qualified as hospital-acquired infections (HAIs) as they take place after day 4. Meanwhile, fatal outcomes occurred during the first 100 days of hospitalization (range 14–89 days), except one occurring on day 270 of hospitalization (Figure 1).

**FIGURE 4:**
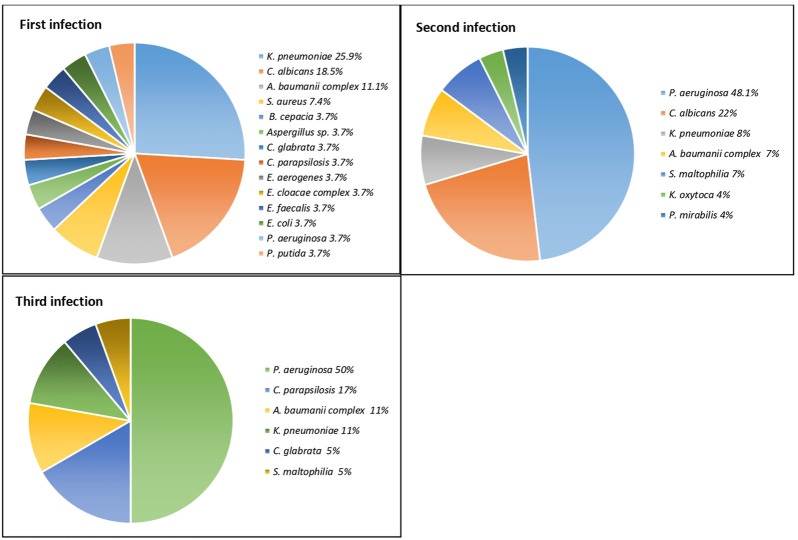
Microorganisms isolated during the three coinfection events. All *Klebsiella pneumoniae* strains belonging to the ssp. *pneumoniae*.

The number of co-infecting microorganisms varies from 2 (nine patients) to 8 (one patient). Four and five coinfecting microorganisms were isolated from six and four patients, respectively, while two patients presented six co-infecting microorganisms (Supplementary Table S1). The most common microorganism isolated from the first infection was *Klebsiella pneumoniae* ssp. *pneumoniae* with seven isolates (25.9%), followed by *Candida albicans* with five isolates (18.5%) (Figure 4; Supplementary Table S1). Regarding the first infection, in 22 cases, it was classified as a hospital-acquired infection, with only those of patients 6 (*K. pneumoniae*), 8 (*Burkholderia cepacia*), 20 (*Staphylococcus aureus*), 25 (*S. aureus*), and 27 (*Klebsiella aerogenes*) being acquired in the community.

In the case of the second infection, *P. aeruginosa* (*n* = 13, 48.1%) was the common pathogen. *Candida albicans* (*n* = 6, 22.2%), *K. pneumoniae* ssp *pneumoniae* (*n* = 4, 15%), and the *Acinetobacter baumannii complex* (*n* = 3, 11%) were also isolated, among other organisms (Figure 3; Supplementary Table S1).

A total of 19 patients presented with a posterior third infection, where *P. aeruginosa* was the microorganism most often identified, with 10 isolates (53%). *Candida parapsilosis* (16%) was observed in three samples and the *A. baumanii complex* (11%) in two samples (Figure 3; Supplementary Table S1).

**FIGURE 5:**
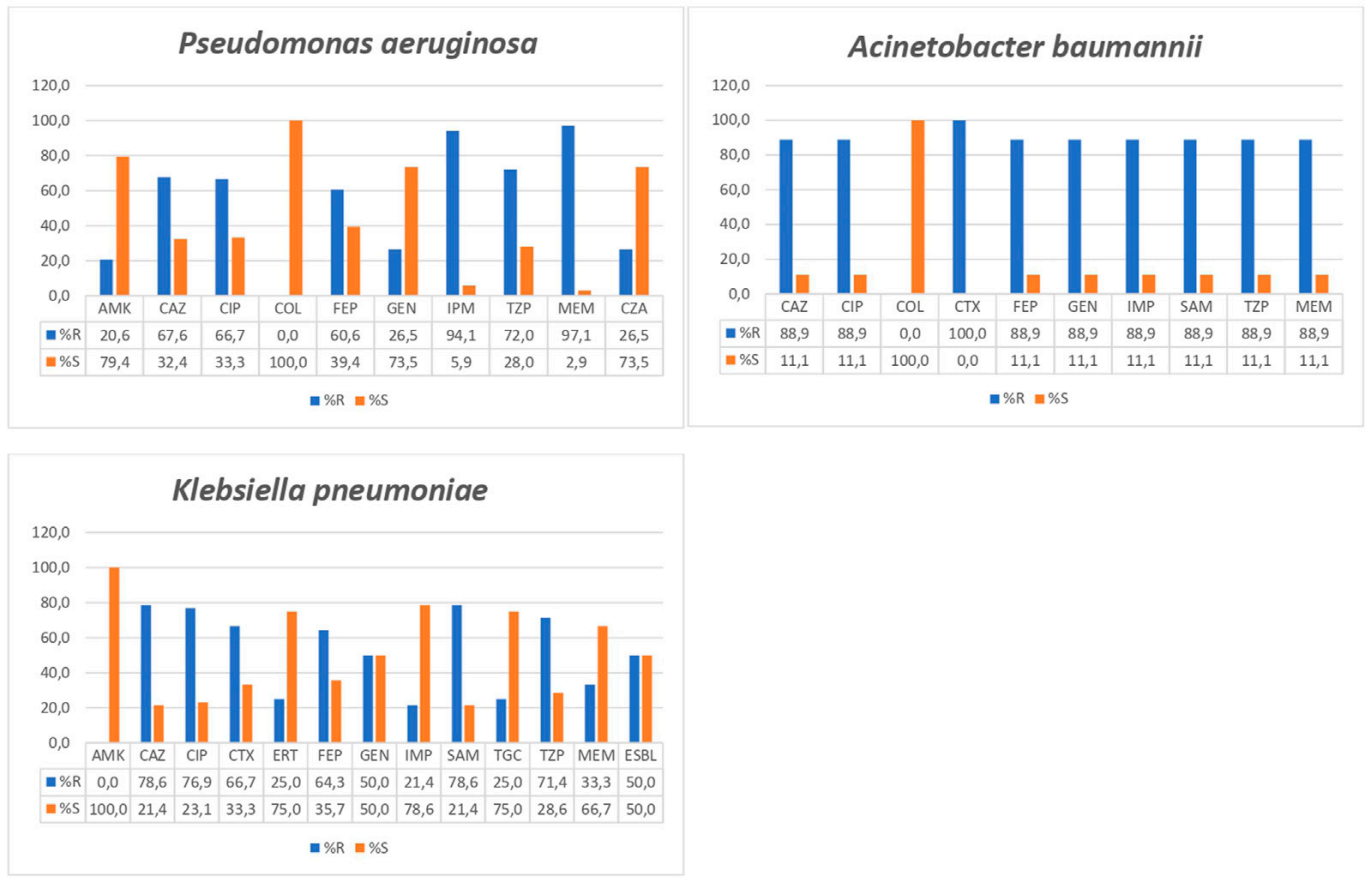
Antimicrobial resistance levels in *Pseudomonas aeruginosa*, *Acinetobacter baumannii*, and *Klebsiella pneumoniae.* CTX, cefotaxime; CAZ, ceftazidime; FEP, cefepime; SAM, ampicillin plus sulbactam; TZP, piperacillin plus tazobactam; IMP, imipenem; MEM, meropenem; ERT, ertapenem; GEN, gentamicin; AMK, amikacin; CIP, ciprofloxacin; TGC, tigecycline; COL, colistin; ESBL, extended spectrum β-lactamases.

As a rule, all microorganisms presented high levels of resistance to tested antimicrobial agents, with the notable exception of colistin. In this sense, all *P. aeruginosa* and *A. baumannii* isolates showed susceptibility to colistin. Regarding the most common microorganisms, *P. aeruginosa* showed levels of resistance to imipenem and meropenem that reached 94.1% and 97.1%, respectively. *K. pneumoniae* presented 78.6% resistance to ceftazidime and ampicillin plus sulbactam and 76.9 to ciprofloxacin, while all isolates were susceptible to amikacin and 50% have extended-spectrum β-lactamases (ESBLs). *A. baumannii* showed the most alarming levels of antimicrobial resistance, showing levels of resistance of 88.9% to all antimicrobial agents tested, except for colistin (Figure 5).

Regarding *Candida* spp., *C. albicans* were fully susceptible to all tested antifungal agents (amphotericin B, caspofungin, fluconazole, micafungin, and voriconazole). Meanwhile, *C. glabrata* were resistant to caspofungin. One *C. parasilapsis* isolate was resistant to both amphotericin B and voriconazole, with no data regarding other antifungal agents.

Among Enterobacteriales, the most noticeable was the presence of resistance to third- and fourth-generation cephalosporins observed in *E. coli*, *Salmonella enterica*, *Enterobacter cloacae*, and one *Proteus mirabilis*, with this later also presenting resistance to carbapenems and ampicillin plus sulbactam. Resistance to fluoroquinolones was observed in *E. coli*, and two out of three *P. mirabilis*, while *S. enterica* presented intermediate susceptibility. Resistance to aminoglycosides was only observed among *P. mirabilis*, with one isolate being amikacin-resistant and the other isolate showing resistance to gentamicin. Finally, the *K. oxytoca* isolate only showed resistance to ampicillin plus sulbactam and piperacillin plus tazobactam, while the *K. aerogenes* isolate was susceptible to all tested antimicrobial agents. Colistin was not tested in these microorganisms.

Regarding other microorganisms, one out of four *Stenotrophomonas maltophilia* species was resistant to cotrimoxazole. All *S. aureus* isolates were susceptible to aminoglycosides, fluoroquinolones, nitrofurantoin, linezolid, quinupristin–dalfopristin, and vancomycin, with two isolates showing resistance to erythromycin and one isolate being resistant to tetracycline. Meanwhile, *E. faecium* was susceptible to nitrofurantoin, linezolid, and vancomycin with all isolates being resistant to quinupristin–dalfopristin, and two of them also being resistant to fluoroquinolones, erythromycin, streptomycin, and tetracycline; one of these isolates was also resistant to gentamicin.

Meropenem (23 patients) and vancomycin (17 patients) were the antibiotics more frequently included in the treatment of present patients (Supplementary Table S1).

## 4 Discussion

COVID-19 has scourged public health in past years, causing more than 6.4 million deaths (World Health Organization, 2022). Health systems in high-income countries were challenged by the pandemic, which led to long waiting and hospital admission delays (Puerta et al., 2022), with the scenario being significantly worsened in low- and middle-income countries due to resource limitations (Maguiña Vargas, 2020; Bhatt et al., 2022). Although the presence of co-infections in COVID-19 patients has been reported (Feldman and Anderson, 2021; Vijay et al., 2021), the present series is focused on the particular scenario of multiple bacterial/fungal co-infections in patients admitted because of SARS-CoV-2 infection.

Different reports have shown that COVID-19 patients may be co-infected by other microorganisms (Sreenath et al., 2021; Vijay et al., 2021; Kurra et al., 2022). In the present series of patients co-infected by SARS-CoV-2 and multiple microorganisms, the rate of fatal outcomes was 25.9% (seven patients). Other studies have shown fatality rates ranging from 9.3% to 56.7%, with differences in underlying factors, such as the date of infection, higher mortality during the peak of infections, and the severity score of the patients (Finelli et al., 2021; Macedo et al., 2021; Vijay et al., 2021)**.** Due to the differences between the immune response to COVID-19 and other microorganisms, the presence of concomitant bacterial/yeast infections would most likely worsen the clinical presentation and severity of COVID-19 (Brodin, 2021). In this sense, the presence of bacterial co-infections in COVID patients has been associated with high mortality rates (Sreenath et al., 2021; Vijay et al., 2021; Kurra et al., 2022).

In agreement with other series of COVID-19 patients, the number of men was higher than that of women (Docherty et al., 2020; Popov et al., 2020; Vijay et al., 2021). However, although not significant, probably due to sample size limitations, fatal outcomes were higher among women. This differs from the most common descriptions, which report high mortality rates among men (Grasselli et al., 2020). It is of note that Vijay et al. (2021), analyzing fatal outcomes in COVID-19 patients after secondary infections, showed similar rates of fatal outcomes among men and women (55% vs. 56%). Although admission after the COVID-19 diagnosis of women was delayed relative to that of men, this is mostly related to the presence of a patient who was admitted 38 days after diagnosis. As no other substantial difference was observed, no specific reason may be adduced to explain this finding, The delay between the diagnosis of COVID-19 and hospital admission was 7 days, longer than that described in other studies, in which patients arrived at the hospital approximately 4 days after the onset of symptoms (Docherty et al., 2020). This finding may be related to the frequent self-medication described among patients with COVID-19 in the country, resulting in a delay in the arrival of patients to the hospital. This delay, at the same time, may lead to an aggravation of patient status (Zavala-Flores and Salcedo-Matienzo, 2020). In this sense, as a mean, those patients with a fatal outcome arrived at the hospital 10.6 days after the diagnosis of COVID-19, instead of 5.4 days of patients who are alive. Unfortunately, no data on previous self-medication were available.

The most common samples were from the respiratory tract, and this type of sample, together with blood, is among the most common sources of co-infecting microorganisms in patients with COVID-19 (Westblade et al., 2021). Regarding comorbidities, several of those described in the present series (obesity, hypertension, and cancer malignant) have often been reported in the literature, being associated with a poor prognosis (Docherty et al., 2020; Richardson et al., 2020). Meanwhile, the presence of a concomitant case of tuberculosis is not a surprising fact in Peru because the disease remains frequent in the area, with COVID-19 probably affecting the description of new cases (Garro Nuñez, 2021).

In our population, patients with up to eight different bacterial/fungi co-infections were observed. In five patients, the first co-infection was reported between 0 and 2 days, and all remaining infections were classified as HAIs. This finding agrees with the nature of most isolated microorganisms, most of them recognized as nosocomial pathogens, as well as with the high levels of antimicrobial resistance observed, typical of HAIs (Vijay et al., 2021).

Unlike the present series, in which the shorter hospitalization period was 14 days, it is reported that patients with severe COVID-19 ended up hospitalized with a median stage of 3–15 days (Vijay et al., 2021). However, longer stays have been widely described (Elhadi et al., 2021). Notably, mechanical ventilation is one of the most common causes of HAIs in ICU patients, with reports arriving at 90% of total HAIs (Cummings et al., 2020; Fumagalli et al., 2022). Consistent with this risk factor, a supplement of oxygen was needed for 26 patients, all but 1.

As mentioned previously, in agreement with the presence of superinfections acquired within the hospital environment, most microorganisms were typical nosocomial pathogens, such as *P. aeruginosa, A. baumannii* complex, or *S. maltophilia*, among others (Vijay et al., 2021). It should be noted that high levels of antimicrobial resistance, as well as the concomitant (or sequential) presence of more than one microorganism, lead to the use of a large number of antimicrobial agents. Although the use of antibacterial agents was needed to treat these co-infections, the use of antimicrobial agents in patients with COVID-19 has been reported to largely exceed the right amounts needed (Getahun et al., 2020). This finding has an impact on bacterial populations as a selective pressure that favors the survival and selection of microorganisms that show high levels of antimicrobial resistance, which, in turn, impairs antibacterial treatments (Ruiz, 2021)**.** In a country such as Peru, with high levels of antibacterial resistance (Levy-Blitchtein et al., 2018; Barrientos-Young et al., in press; Flores-Paredes et al., 2021), and *de facto* over-the-counter access to antibacterial agents (Zavala-Flores and Salcedo-Matienzo, 2020), it may contribute to the selection of practically untreatable pathogenic microorganisms.

The high levels of antimicrobial resistance observed, including the lack of usefulness of all antibacterial agents (except colistin) to combat infections of the *A. baumannii* complex, strongly highlight the need to install strict control measures in the use of antimicrobial agents in the country, as well as the urge to explore new alternatives to the current antibiotic schedule. Regarding colistin, the only antimicrobial agent tested that shows 100% activity against *A. baumannii* and *P. aeruginosa,* the description of colistin-resistant pathogenic microorganisms in the area is of concern (Naomi-Matsuoka et al., 2020).

There are some limitations to this study, including the sample size. However, the present data show a relevant number of patients co-infected by a series of hospital-acquired multidrug-resistant pathogens that impaired patient outcome. Notably, patients were diagnosed at the beginning of 2021 in a moment of high COVID pressure in the country, resulting in the possible lack of several data on records.

This study highlights the presence of multiple bacterial co-infections in a scenario of hospital stress, and this highlights the need to maintain and reinforce prevention measures to minimize nosocomial infections at times of maximum hospital saturation.

The clinical characteristics of these patients would increase the evidence of co-infection by multiple microorganisms, the management, and better knowledge in a scenario of COVID-19. Furthermore, early diagnosis and establishment of bacterial co-infections and antibiotic susceptibility profiles are also relevant to avoid unnecessary treatment and ensure adequate treatments when needed.

## Data Availability

The original contributions presented in the study are included in the article/Supplementary Material; further inquiries can be directed to the corresponding authors.
